# GWAS and Post-GWAS High-Resolution Mapping Analyses Identify Strong Novel Candidate Genes Influencing the Fatty Acid Composition of the *Longissimus dorsi* Muscle in Pigs

**DOI:** 10.3390/genes12091323

**Published:** 2021-08-26

**Authors:** Jae-Bong Lee, Yong-Jun Kang, Sang-Geum Kim, Jae-Hoon Woo, Moon-Cheol Shin, Nam-Geon Park, Byoung-Chul Yang, Sang-Hyun Han, Kang-Min Han, Hyun-Tae Lim, Youn-Chul Ryu, Hee-Bok Park, In-Cheol Cho

**Affiliations:** 1 Korea Zoonosis Research Institute, Jeonbuk National University, Iksan 54531, Korea; jblee@jbnu.ac.kr; 2 Subtropical Livestock Research Institute, National Institute of Animal Science, RDA, Jeju 63242, Korea; yjkang1201@korea.kr (Y.-J.K.); jimibong@daum.net (S.-G.K.); delpire@korea.kr (J.-H.W.); shinemoon@korea.kr (M.-C.S.); parkng@korea.kr (N.-G.P.); bcyang@korea.kr (B.-C.Y.); 3 Species Restoration Technology Institute, Korea National Park Service, Yeongju 36015, Korea; hansh04@knps.or.kr; 4 Department of Pathology, Dongguk University Ilsan Hospital, Goyang 10326, Korea; kiekie53@hanmail.net; 5 Department of Animal Science, College of Agriculture and Life Sciences, Gyeongsang National University, Jinju 52828, Korea; s_htim@gnu.ac.kr; 6 Institute of Agriculture and Life Science, Gyeongsang National University, Jinju 52828, Korea; 7 Division of Biotechnology, Jeju National University, SARI, Jeju 63243, Korea; ycryu@jejunu.ac.kr; 8 Department of Animal Resources Science, Kongju National University, Yesan 32439, Korea

**Keywords:** GWAS, high-resolution mapping, *longissimus dorsi* muscle, fatty acid profile, QTL, *GAS7*, *MYH2*, *MYH3*, human health, pork quality

## Abstract

Fatty acid (FA) composition is one of the most important parameters for the assessment of meat quality in pigs. The FA composition in pork can also affect human health. Our aim was to identify quantitative trait loci (QTLs) and positional candidate genes affecting the FA profile of the *longissimus dorsi* muscle in a large F2 intercross between Landrace and Korean native pigs comprising 1105 F2 progeny by genome-wide association studies (GWAS) and post-GWAS high-resolution mapping analyses. We performed GWAS using the PorcineSNP60K BeadChip and a linear mixed model. Four genome-wide significant QTL regions in SSC8, SSC12, SSC14, and SSC16 were detected (*p* < 2.53 × 10^−7^). Several co-localizations of QTLs in SSC12 for oleic acid, linoleic acid, arachidonic acid, monounsaturated FAs, polyunsaturated FAs, and the polyunsaturated/saturated FA ratio were observed. To refine the QTL region in SSC12, a linkage and linkage disequilibrium analysis was applied and could narrow down the critical region to a 0.749 Mb region. Of the genes in this region, GAS7, MYH2, and MYH3 were identified as strong novel candidate genes based on further conditional association analyses. These findings provide a novel insight into the genetic basis of FA composition in pork and could contribute to the improvement of pork quality.

## 1. Introduction

FA composition plays an important role in the evaluation of pork quality because it can contribute to sensorial, nutritional, and functional factors. For example, FA composition controls the oxidative stability of pork, which in turn affects meat color and flavor [1]. Linoleic acid (an omega-6 FA) and α-linolenic acid (an omega-3 FA) are regarded as essential fatty acids because they cannot be synthesized in mammals [2]. In addition, the ratio of unsaturated FA (UFA) and saturated FA (SFA) levels in membrane fluidity and cell–cell interactions has been implicated in various types of human diseases, such as obesity [3], hypertension [4], diabetes [5], and cancer [6]. Hence, FA composition not only plays a crucial role in the quality of pork but is also highly relevant to human health. Recently, multiple genomic approaches have been applied to elucidate the genetic architecture of FA composition in pork, such as GWAS using imputed sequence data [7], expression QTL study [8], and RNA-seq analyses [9,10].

Despite their slow growth rate, Korean native black pigs (KNPs), which inhabit Jeju Island, are renowned for their meat quality, which fulfills current consumer requirements, such as reddish meat color, white-colored fat, solid fat structure, and good marbling [11,12]. Concerning the FA composition of KNP, it was reported that the total UFA contents were higher in KNPs than in Western crossbred (i.e., (Landrace × Yorkshire) × Duroc) pigs [13]. Although Park et al. [14] conducted genome-wide linkage analyses and identified several QTLs affecting the FA profile using an F_2_ intercross between Landrace pigs and KNPs, detailed studies on the individual major genes that are responsible for the FA profile of KNP are still limited. Hence, we conducted a GWAS in combination with post-GWAS high-resolution mapping analyses to reveal individual positional candidate gene(s) for the FA profile traits in the *longissimus dorsi* muscle using an F_2_ intercross between Landrace and KNP pigs.

## 2. Materials and Methods

### 2.1. Ethics Statement

All experimental procedures were conducted according to national and institutional guidelines and approved by the Ethical Committee of the National Institute of Animal Science, Republic of Korea (Approval number (date): 2014-095 (6 August 2014)).

### 2.2. Animals

A total of 1105 animals of the F_2_ resource population (568 male and 537 female) were established by crossing purebred Landrace pigs and purebred KNPs (LK cross). Nineteen KNPs were crossed with 17 Landrace pigs, generating 91 animals in the F_1_ generation. Subsequently, F_1_ progeny were intercrossed to generate F_2_ offspring. All experimental procedures for breeding, feeding, and phenotyping took place at the same research farm in the Subtropical Livestock Research Institute of the National Institute of Animal Science, RDA, which is located on Jeju Island. All pigs were managed with the same procedure at farrowing. Male pigs were not castrated. Male and female piglets were weaned at 21 days of age. All F_2_ experimental animals were slaughtered in the same commercial abattoir. This population has previously been used in linkage and association studies for mapping QTLs affecting growth [15,16], meat quality [14,17], serum traits [18], and hematological traits [19,20]. A detailed description of the LK cross can be found in [19].

### 2.3. Genotype

All experimental animals in the LK cross were genotyped for 62,163 single nucleotide polymorphism (SNP) markers using the Porcine SNP 60K BeadChip (Illumina Inc., San Diego, CA, USA). Genomic DNA was extracted from the blood using a standard sucrose–proteinase K method. Quality control and filtration of genotyped SNP markers were performed using the PLINK program (ver. 1.90) [21]. The genotypes were filtered according to minor allele frequency (<5%), genotype call rate (<90%), and a *p*-value of the χ^2^-test for Hardy–Weinberg equilibrium errors (≤0.000001). A total of 39,485 SNP markers on 18 autosomes remained after filtration and quality control.

### 2.4. Phenotype

*Longissimus dorsi* muscle was sampled from the F_2_ intercross for FA composition data. According to the procedure of Folch et al. [22], intramuscular lipids in each *longissimus dorsi* muscle sample were separated using chloroform–methanol (2:1, *v/v*), and lipid extracts were transformed to FA methyl esters using Morrison and Smith’s method [23]. The separation and quantification of the FA methyl esters were conducted using a gas chromatograph (6890N, Agilent Technologies, Germany). Table 1 shows the descriptive statistics and heritability for the FA traits. Several of the FA profile traits showed significant deviation from normality. Prior to the statistical genetic analyses, these data were transformed using the natural logarithm (i.e., C18:2 and PUFAs) or the square root (i.e., C20:1 and C20:4) to remove skewness.

### 2.5. Estimation of Heritability and GWAS for Mapping QTLs 

The ASReml program (ver. 4.0) was used to estimate the heritability of each FA trait recorded in this study (VSN International, UK), and the following linear mixed model was used for analysis:

**y = Xb + Zu + e** (1)

where **y** is the vector of FA phenotypes and **b** is the vector of fixed effects including the intercept, sex, batch, and carcass weight; **u** is the vector of random additive effects following a normal distribution **u**~N(0, **A**σ_a_^2^), in which **A** is the numerator relationship matrix based on pedigree and σ_a_^2^ is the additive genetic variance; **e** is the vector of random residual following a normal distribution **e**~N(0, **I**σ_e_^2^), in which **I** is the identity matrix and σ_e_^2^ is the residual variance; and **X** and **Z** are the incidence matrices for **b** and **u**.

A single-marker association analysis was performed to identify QTLs for FA profile traits using the genome-wide efficient mixed-model association (GEMMA) program (ver. 0.94.1) [24]. This approach adjusts the familial relatedness within the F_2_ intercross during the GWAS. The following linear mixed model was used to assess the association between SNP markers and FA profile traits:

**y = Xb + Z_1_a + Z_2_u + e** (2)

where **y** is the vector of FA phenotypes and **b** is the vector of fixed effects including the intercept, sex, batch, and carcass weight; **a** is the vector of fixed effect of SNP marker; **u** is the vector of random additive effects following a normal distribution **u ~** N(0, **G**σ_g_^2^), in which **G** is the genomic relationship matrix that was constructed using the 39,485 SNP markers and σ_g_^2^ is the additive genetic variance; **e** is the vector of random residual following a normal distribution **e**~N(0, **I**σ_e_^2^), in which **I** is the identity matrix and σ_e_^2^ is the residual variance; **X** is the incidence matrix of **b**; and **Z_1_** and **Z_2_** are the incidence matrices for **a** and **u**.

To address multiple comparison issues of the GWAS, a Bonferroni-adjusted significance threshold (i.e., 0.01/39,485 SNP markers, *p* < 2.53 × 10^−7^) was calculated for the LK cohort. Based on the significance threshold, a GWAS was performed chromosome by chromosome. The confidence interval of these QTL regions was determined using two SNPs (upper boundary and lower boundary) having nominal *p*-values lower than the significance threshold (*p* < 2.53 × 10^−7^) in the neighborhood of the top-ranked significant SNP. The % proportion of phenotypic variance (%VAR_SNP_) explained by the top-ranked SNP was computed using the following equation:

%VAR_SNP_ = [2*p*(1 − *p*)*a*^2^/σ_p_^2^ ] × 100 (3)

where *p* is the minor allele frequency of the marker, *a* is the estimated allelic effect of the marker, and σ_p_^2^ is the phenotypic variance component for each FA profile trait. The phenotypic variance component (σ_p_^2^) was estimated using the GCTA program (ver. 1.93.2) [25]. 

### 2.6. Joint Linkage and Linkage Disequilibrium (LALD) Analysis for High-Resolution QTL Mapping

High-resolution mapping of the most significant QTL detected by GWAS was performed by jointly integrating both linkage (LA) and linkage disequilibrium (LD) using a haplotype-based approach. First, the CRIMAP program (ver. 2.507), developed by Evans and Maddox (URL: http://www.animalgenome.org/bioinfo/tools/share/crimap, accessed on 7 November 2017), was used to establish the genetic linkage map of the chromosome harboring the most significant QTL detected by GWAS. Second, founder haplotypes found in the parental pigs (i.e., Landrace and KNP) were reconstructed using the DualPHASE program (ver. 1.1) [26], which integrates LA information obtained at the family level and LD information obtained at the population level in a Hidden Markov Model framework. A total of 20 founder haplotype clusters (*K* = 20) were used. Third, the estimated haplotypes were incorporated into a linear mixed model including fixed (i.e., sex, batch, and carcass weight) and random (i.e., the 20 effects of founder haplotypes and animal effects) effects and random residual error terms to perform high-resolution mapping of QTL analysis using the QxPAK program (ver. 5.05) [27]. The confidence interval of the fine-mapped QTL was established using the 1-LOD drop method [28].

### 2.7. Further Conditional Association Analyses for Positional Candidate Gene Identification

Using the GCTA program (ver. 1.93.2), conditional association analyses were performed to further refine the critical region identified by LALD analyses [29]. This was achieved using a multiple regression frame to test the hypothesis of the presence of QTL at a particular DNA marker conditioned on selected markers (i.e., key markers) as cofactors [30,31]. Prior to conditional analysis, a stepwise regression was performed to select key SNP marker(s) using the GCTA-slct procedure. Subsequently, the selected key SNP markers were incorporated into a multiple regression based on a GCTA-cojo model as cofactors, and the effect of other SNP markers in the critical region was evaluated to determine whether additional independent association signals were present. A threshold of *p* < 0.0005 was applied to determine the statistical significance of the conditional analyses; this corresponded to the Bonferroni adjustment threshold for the critical region by LALD analysis.

### 2.8. Positional Candidate Gene Analyses

The gene list and map position in each QTL was extracted from the NCBI database release 85 based on *Sus scrofa* 11.1 assembly. A list of genes and the map position in each QTL region was obtained from the NCBI database. If locus information was not available in the NCBI database, the ENSEMBL database based on *Sus scrofa* 11.1 assembly was used. Comparative analysis of QTL locations was conducted using the Animal QTLdb (URL: https://www.animalgenome.org/cgi-bin/QTLdb/index, accessed on 8 January 2021).

### 2.9. Haplotype Reconstruction and Haplotype-Based Association Analysis

Pigs in the F_2_ generation were used to construct haplotypes at the loci of interest using the SNP markers described in Supplementary Table S1. The Beagle program (ver. 3.3.0.) was applied to construct the haplotypes [32]. The haplotype data of the F_2_ pigs were used to perform haplotype-based (diplotype) association analysis. The effect of haplotypes on FA traits was evaluated by the following general linear model (GLM) using the MINITAB program (ver. 14, MINITAB, State College, PA, USA):

Y_ijkl_ = μ + S_i_ + B_j_ + D_k_ + bCW_ijkl_ +e_ijkl_ (4)

where Y_ijkl_ is the phenotypic value, μ is the overall population mean, S_i_ is the fixed effect of sex, B_j_ is the fixed effect of batch, D_k_ is the fixed effect of diplotype (Supplementary Table S1), b is a regression coefficient, CW_ijkl_ is covariate for the carcass weight, and e_ijkl_ is the residual term of the model. To determine the statistical significance of the GLM analyses, a threshold of *p* < 0.01 was used.

## 3. Results and Discussion

Basic descriptive statistics and heritability of the 18 phenotypic records of FA traits in the *longissimus dorsi* muscle in the F_2_ pigs are described in Table 1. The most abundant FA was C18:1, followed by C18:0, C18:2, and C16:1 in the F_2_ population. The average estimate of heritability for the 19 FA composition traits was 0.396, which indicates that the contribution of genetic effects to phenotypic variation in FA composition traits is substantial. 

### 3.1. GWAS Detected a Major QTL for FA Composition in SSC12 in the LK Cross

**Figure 1:**
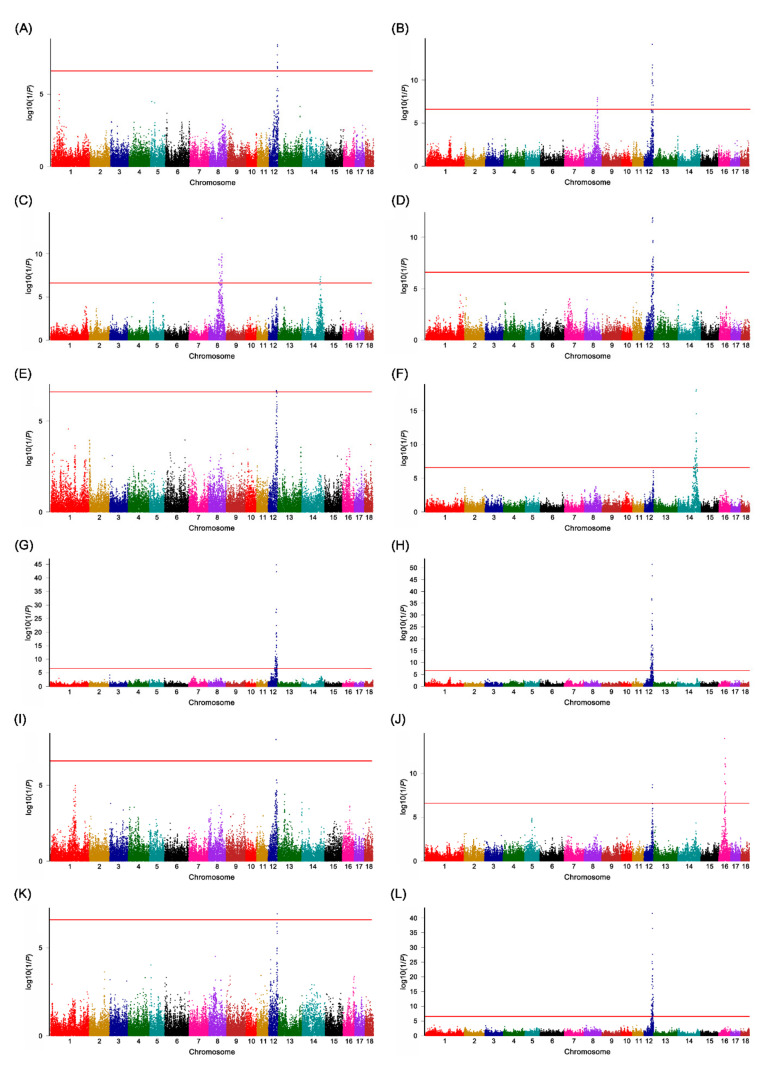

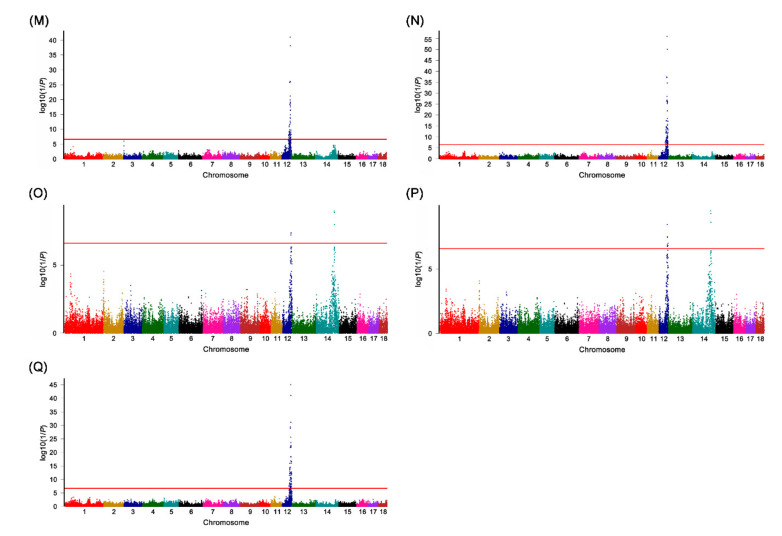
Manhattan plots of the GWAS for the fatty acid composition traits in the LK cross. The y-axis shows the log_10_(1/*p*-value), and the x-axis shows the genomic map positions of the SNP markers on the pig autosomes. The genome-wide significance threshold value is 6.60, which equals a Bonferroni correction of 1% (represented by the red horizontal lines). (**A**) Manhattan plot for C12:0; (**B**) Manhattan plot for C16:0; (**C**) Manhattan plot for C16:1; (**D**) Manhattan plot for C17:0; (**E**) Manhattan plot for C17:1; (**F**) Manhattan plot for C18:0; (**G**) Manhattan plot for C18:1; (**H**) Manhattan plot for C18:2; (**I**) Manhattan plot for C18:3; (**J**) Manhattan plot for C20:0; (**K**) Manhattan plot for C20:1; (**L**) Manhattan plot for C20:4; (**M**) Manhattan plot for MUFAs; (**N**) Manhattan plot for PUFA; (**O**) Manhattan plot for SFA; (**P**) Manhattan plot for UFA; (**Q**) Manhattan plot for P/S ratio. The Manhattan plots show the identification of the major QTL for FA profile traits in SSC12.

To investigate the genetic foundation underlying the FA traits of the *longissimus dorsi* muscle in pigs, we used a large F_2_ intercross between Landrace pigs and KNP comprising 1105 F2 offspring. Using this cohort, GWAS detected 22 genome-wide significant QTLs (*p* < 2.53 × 10^−7^) for the 17 FA traits located on pig chromosome 8 (SSC8), SSC12, SSC14, and SSC16 (Table 2, Figure 1, Supplementary Figure S1). These QTLs explained 4.4 to 29.4% of the phenotypic variance in FA phenotypes (Table 2). 

In SSC8, we mapped the QTL regions for C16:0 and C16:1 (Table 2). Previously reported QTLs for C16:0 and C16:1 were colocalized to the identified QTL in SSC8 in this study [14] [33,34]. This QTL region harbors a known positional candidate gene: the ELOVL6 gene. The ELOVL6 (elongation of very-long-chain fatty acid protein 6) gene is located at 111.75-Mb. This gene plays a critical role in assembling carbons, catalyzing 16-carbon FA to form 18-carbon FA [35]. Corominas et al. [36] reported that the level of expression of the ELOVL6 gene was associated with C16:0 and C16:1 contents and the elongation activity ratio in pig muscle.

In SSC14, QTLs for C16:1, C18:0, SFAs, and UFAs were identified (Table 2). Formerly reported QTLs for the four FA traits were mapped to the same region of identified QTL in SSC14 in this study [14,34,37,38]. The QTLs for C18:0, SFA, and UFA contain the SCD gene. The SCD (stearoyl-CoA desaturase) gene is located at 111.46-Mb. SCD plays a pivotal role in regulating the ratio of C18:0 to C18:1. This enzyme also controls the ratio of C16:0 to C16:1 [39]. We previously reported that the SCD gene has a considerable effect on the FA profile in this F_2_ cohort [40]. 

In SSC16, a QTL for C20:0 was detected (Table 2). Formerly identified QTLs for C20:0 were colocalized to the QTL identified in SSC16 in this study [14,41]. The QTL region includes the ELOVL7 gene. The ELOVL7 (elongation of very-long-chain fatty acid protein 7) gene is located at 39.46 Mb. This gene encodes an elongase that is involved in the catalysis of very-long-chain fatty acids, including C20:0 [42]. Thus, the ELOVL7 gene can be regarded as a strong positional candidate gene for C20:0 in this QTL.

In SSC12, we identified genomic regions enriched in QTLs for the trait of interests (Figure 1, Table 2). In particular, the QTLs for C18:1, C18:2, C20:4, MUFAs, PUFAs, and the P/S ratio showed much lower *p*-values than the other 9 QTLs. These QTLs span from 42.14 to 59.56 Mb (17.42 Mb region) in SSC12 and contain 344 annotated genes. However, the QTL region is too large to pinpoint positional candidate genes for the FA traits in SSC12. 

### 3.2. Post-GWAS LALD Mapping Narrowed down the QTL Confidence Interval to a 749.1 kb Critical Region in SSC12 

**Figure 2:**
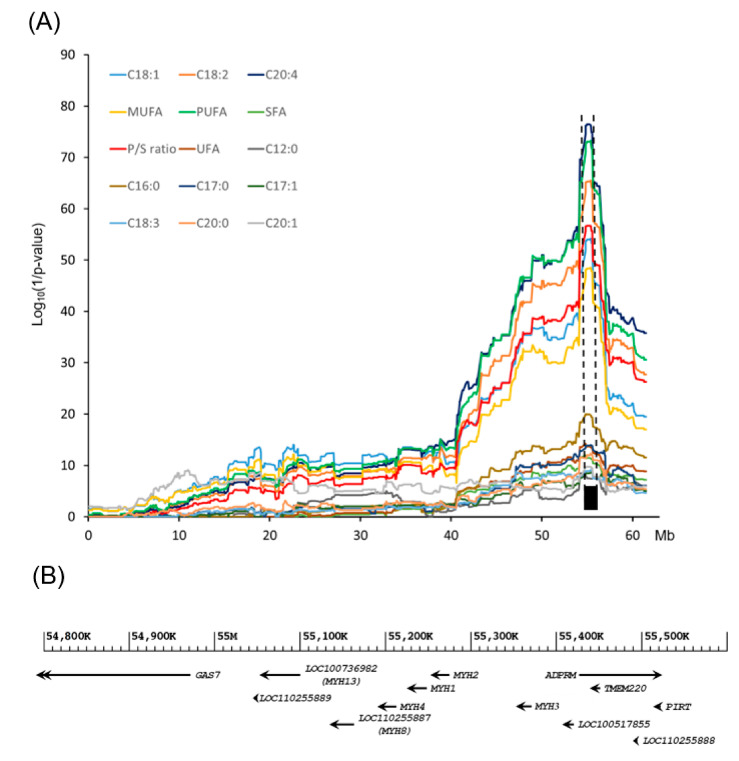
Post-GWAS high-resolution mapping of a QTL that influences FA composition traits of the *longissimus dorsi* muscle of the LK cross. (**A**) LALD mapping results in SSC12 for FA traits. The y-axis shows the log_10_(1/*p*-value), and the x-axis shows the genomic map positions of the SNP markers in SSC12. The width of the black rectangle represents 1-LOD drop confidence interval corresponding to the 749.1 kb region in SSC12 (12: 12:54,812,172-55,561,243). (**B**) Ten NCBI protein-coding genes are located within the 749.1 kb region associated with the FA composition traits. Gene names in parentheses were annotated by our previous study [12].

We conducted a joint linkage and linkage disequilibrium (LALD) mapping to narrow down the confidence intervals (CIs), including positional candidate genes (Figure 2). A 1-LOD drop method was applied to estimate the CI of the QTLs in SSC12 for 15 FA traits [28]. The longest CI was 8.02 Mb, while the shortest CI was 0.749 Mb. The 0.75 Mb region was shared across the 15 QTLs for FA traits in SSC12 (Supplementary Figure S2). Thus, we defined the CI of the QTLs for FA traits as a 749.1 kb region (12:54,812,172-55,561,243 bp). This critical region overlaps with the previously reported 718.4 kb region (12:54,842,795-55,561,243) affecting the intramuscular fat (IMF) content in *longissimus dorsi* in the LK cross [12]. To the best of our knowledge, this critical region in SSC12 was revealed for the first time for muscle FA traits in pigs in this study. Recently, a comprehensive GWAS for FA profile traits was reported using multiple pig populations [7]. This study also detected a significant association of SNPs on SSC12 with FA composition traits. However, the location of the SNPs was not overlapped with the critical region that we revealed in this study. This critical 749.1 kb region in SSC12 encompasses 10 annotated genes (i.e., *GAS7*, *MYH13*, *MYH8*, *MYH4*, *MYH1*, *MYH2*, *MYH3*, *ADPRM*, *TMEM220,* and *PIRT*) with 19 SNP markers in the *Sscrofa* 11.1 genome dataset.

As shown in Figure 2, the LALD profiles for C18:1, C18:2, C20:4, MUFAs, PUFAs, and the P/S ratio showed a cluster with an extremely high log_10_(1/*p*-value) compared to those of the other nine traits. In addition, the CIs of the six traits defined by the LALD analysis were narrower than those of the other 9 traits (Supplementary Figure S2). Thus, we focused on these six FA traits for further high-resolution mapping.

### 3.3. Post-GWAS Conditional Association Analysis of the 749.1 kb Critical Region Identified Strong Candidate Genes 

Prior to conditional association analysis, we performed a stepwise regression analysis to select key SNP marker(s) among the 19 SNP markers in the 749.1 kb critical region using the GCTA-slct procedure. The selected key SNP was used as a cofactor for subsequent conditional analysis in the critical region. From the variable selection analysis based on stepwise regression, *GAS7:g.18482 T>C* (12:54,956,054) was selected as the single key marker across the six FA traits (Table 3, Table 4 and Table 5). In the single-marker association analysis, this marker showed a strong association with C18:1, C18:2, C20:4, MUFAs, PUFAs, and P/S ratio (Table 2, Supplementary Figure S3). Further, we encountered similar evidence of a strong association among adjacent SNP markers in the 749.1 kb critical region because of the comprehensive linkage disequilibrium (LD) structure (Supplementary Figure S3) [43].

To explore the possibility of dissociating association signals in the high LD region, we performed a conditional association analysis by conditioning the key SNP at each of the remaining SNP markers in the 749.1 kb critical region. Except for one SNP (intron ENSSSCG00000044652:g.8526 T>C (12:55,530,321)) showing marginal significance for C18:1 (*p* = 0.0004), no additional significant association signals were detected (Table 3, Table 4 and Table 5). Notably, we could not compute the adjusted *p*-value of three SNP markers (*MYH2:* c. −449935 A>G (12:55,229,376), *MYH2:* c.−22675 T>C (12:55,262,653), and *MYH3*−1805_−1810delCAGTCC (15:55,373,707)) in the critical region for the six FA traits (Table 3, Table 4 and Table 5). A single-marker association study reported that these three SNPs showed extremely high significance. However, it was not possible to statistically resolve the effect of these SNP markers, given that this collinearity originated from the LD structure. The LD correlation (*r*) values were >0.996 between the three SNPs and the key marker (Table 3, Table 4 and Table 5; Supplementary Figure S3); therefore, we constructed haplotypes of the four markers to evaluate the combined effect of the three genes (i.e., *GAS7*, *MYH2*, and *MYH3*) on the FA traits.

### 3.4. Haplotype Construction and Haplotype-Based Association Analysis

**Figure 3:**
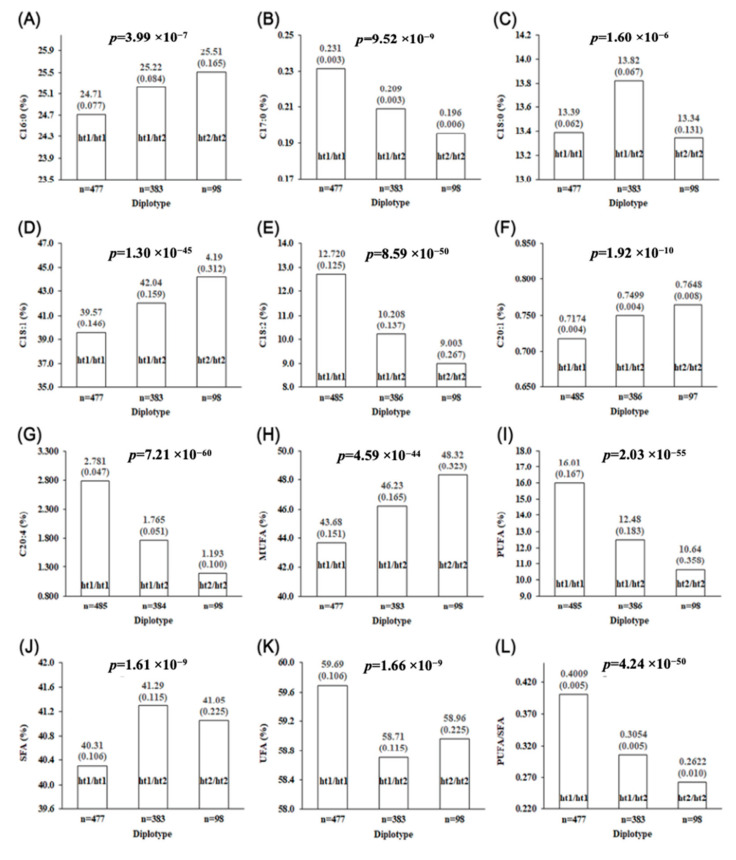
Haplotypic effect of the variants in GAS7, MYH2, and MYH3 genes on FA profile traits of the longissimus dorsi muscle in the LK cross (*p* < 0.01). Each bar represents the least square mean of each diplotype of the FA profile traits. The number in parentheses above each bar represents the standard error. (**A**) Bar graph for C16:0; (**B**) bar graph for C17:0; (**C**) bar graph for C18:0; (**D**) bar graph for C18:1; (**E**) bar graph for C18:2; (**F**) bar graph for C20:1; (**G**) bar graph for C20:4; (**H**) bar graph for MUFAs; (**I**) bar graph for PUFAs; (**J**) bar graph for SFA; (**K**) bar graph for UFA; (**L**) bar graph for P/S ratio.

We constructed haplotypes of the SNP markers of the GAS7, MYH2, and MYH3 genes to evaluate the effect of haplotypes derived from the three genes on the FA traits. The haplotype and diplotype frequencies in the F_2_ pigs are listed in Supplementary Table S1. The results of haplotype-based association analysis indicated that there were significant associations between diplotype and FA traits in F2 progeny (Figure 3). The effects of diplotype on C18:1, C18:2, C20:4, MUFAs, PUFAs, and the P/S ratio showed extremely low *p*-values compared to those of the other of 6 traits (Figure 3). Moreover, some of the data (i.e., C16:0, C17:0, C18:1, C18:2, C20:1, C20:4, MUFAs, PUFAs, and P/S ratio) fit considerably well to an additive model of inheritance (i.e., the genotypic value of heterozygote lies in the middle of the genotypic value of the two homozygotes).

### 3.5. GAS7, MYH2, and MYH3 Were Revealed as Novel Strong Candidate Genes for FA Composition

The FA composition of meat lipids has various effects on meat quality and human health. For example, the proportion of MUFAs, specifically oleic acid (C18:1), was strongly and positively correlated with overall palatability, including flavor, juiciness, and tenderness in beef [44]. Among UFAs, PUFAs have been in the spotlight because of their diverse physiological functions. PUFAs are essential factors in several cellular functions and modulate the physical properties of cell membranes and the gene expression of enzymes involved in triglyceride storage and fatty acid oxidation [45]. Thus, adequate intake of PUFAs in human diets can reduce the risk of several diseases, including cardiovascular diseases [46]. Since the haplotypes of the GAS7, MYH2, and MYH3 genes were reciprocally associated with MUFAs and PUFAs, marker-assisted selection of haplotype1 (ht1) can result in the production of PUFA-enriched pork with low palatability. By contrast, marker-assisted selection of the haplotype2 (ht2) can lead to the production of pork with high MUFAs content. Thus, marker-assisted selection of the ht1/ht2 diplotype could be an immediate approach to simultaneously acquiring the two FA profile characteristics in pork. 

The GAS7 gene encodes growth arrest-specific protein 7, which was initially known for its important roles in neuronal development and is implicated in the regulation of lipid metabolism [47,48]. Yang et al. [49] constructed *GAS7* transgenic (Tg) mice and reported decreased adiposity and unesterified cholesterol in a Tg mouse model. They also suggested a putative involvement of the GAS7 gene in free FA metabolism on the basis of pathway and network analyses. In the case of genes encoding myosin heavy chain (MYH) isoforms, Lim et al. reported effects of SNPs in the *MYH2* locus on muscle fiber characteristics and meat quality including IMF content [50]. Recently, we also reported that a functional regulatory variant of *MYH3* is causatively associated with muscle fiber-type composition and IMF content in the same population used in this study [12]. Myoblasts and adipoblasts originate from the same mesoderm layer in embryos, and many studies on the multipotential capacity of muscle satellite cells to differentiate into myogenic, adipogenic, and osteogenic cells have been conducted. However, knowledge of the implications of MYH isoforms in this transdifferentiation between myoblasts and adipoblasts is still limited [51]. Therefore, further functional studies are required to reveal the potential involvement of MYH isoforms in the transdifferentiation process. Because of the strong genetic correlation between IMF content and FA profile [52], our results uncover the possibility of simultaneous manipulation of muscle FA composition and IMF content by identifying the significant haplotype effect of three genes on the FA profile.

## 4. Conclusions

In this study, we aimed to identify individual positional candidate gene(s) in the FA profile of the *longissimus dorsi* muscle in an intercross between Landrace pigs and KNPs. For this purpose, we conducted a GWAS in combination with post-GWAS high-resolution mapping analyses. The GWAS approaches identified significant QTLs in SSC8, SSC12, SSC14, and SSC16 at the genome-wide level. Relevant concordance was revealed between the QTLs detected in this study and previously reported QTLs for muscle FA traits, mainly in SSC8, SSC14, and SSC16. The QTL in SSC12 has not been well documented for FA traits. The post-GWAS high-resolution mapping analyses of the QTL in SSC12 identified a 749.1 kb critical region, and the GAS7, MYH2, and MYH3 genes were identified as strong candidate genes for FA traits. Furthermore, although we could not elucidate the individual contribution of the three genes to the phenotypic variation of the FA traits because of the extremely high LD, these findings provide new insight into the genetic basis of FA composition in pork and could contribute to the genetic improvement of pork quality.

## Figures and Tables

**Table 1:** Summary of fatty acid composition data from the F2 generation of an intercross between Landrace and Korean native pigs.

Phenotype	Label	^1^ N	Mean	SD	Range	^2^*h*^2^ ± SE
C12:0 (%)	Lauric acid	963	0.096	0.01	0.06–0.16	0.48 ± 0.12
C14:0 (%)	Myristic acid	964	1.58	0.20	0.67–2.68	0.34 ± 0.11
C16:0 (%)	Palmitic acid	959	25.0	1.31	20.8–29.3	0.48 ± 0.12
C16:1 (%)	Palmitoleic acid	965	3.15	0.50	1.53–4.61	0.37 ± 0.10
C17:0 (%)	Margaric acid	957	0.22	0.06	0.01–0.37	0.15 ± 0.07
C17:1 (%)	Margar oleic acid	960	0.25	0.04	0.09–0.45	0.23 ± 0.09
C18:0 (%)	Stearic acid	954	13.6	1.18	8.52–18.5	0.32 ± 0.10
C18:1 (%)	Oleic acid	961	40.9	3.73	27.7–52.1	0.42 ± 0.12
C18:2 (%)	Linoleic acid	976	11.4	3.50	4.50–25.8	0.46 ± 0.12
C18:3 (%)	α-Linolenic acid	964	0.47	0.11	0.22–0.84	0.36 ± 0.11
C20:0 (%)	Arachidic acid	961	0.22	0.04	0.12–0.36	0.49 ± 0.11
C20:1 (%)	Gadoleic acid	975	0.74	0.08	0.40–1.11	0.42 ± 0.11
C20:4 (%)	Arachidonic acid	974	2.19	1.32	0.16–8.85	0.54 ± 0.12
SFA (%)	Saturated fatty acid	965	40.8	2.38	23.4–59.6	0.35 ± 0.11
UFA (%)	Unsaturated fatty acid	965	59.2	2.38	40.4–76.6	0.35 ± 0.11
MUFA (%)	Monounsaturated fatty acid	965	45.1	4.18	27.8–70.8	0.42 ± 0.12
PUFA (%)	Polyunsaturated fatty acid	976	14.1	4.69	5.19–29.9	0.48 ± 0.12
P/S ratio	PUFA/SFA	965	0.35	0.13	0.11–0.82	0.46 ± 0.12

^1^ Number of phenotyped animals; ^2^ heritability of the FA traits.

**Table 2:** Genome-wide significant QTLs for FA traits in an F2 intercross between Landrace.

^1^ SSC	Trait	^2^ N_snp_	^3^ Interval (Mb)	Top SNP	^4^ Position (bp)	^5^*p*-Value	^6^ %Var	Known Candidate Genes
8	C16:0	7	109.8–114.1	*MARC0028605*	113291506	1.15 × 10^−8^	6.4	*ELOVL6*
8	C16:1	31	85.3–114.8	*MARC0025408*	112754574	8.07 × 10^−15^	10.3	
12	C12:0	8	54.3–56.1	*MARC0017535*	55856415	3.94 × 10^−9^	6.1	
12	C16:0	23	50.1–59.6	*M1GA0017062*	54956054	7.31 × 10^−15^	8.7	
12	C17:0	27	50.1–58.9	*M1GA0017062*	54956054	1.25 × 10^−12^	8.9	
12	C17:1	5	54.9–56.4	*M1GA0017062*	54956054	2.09 × 10^−7^	4.8	
12	C18:1	71	44.7–59.6	*M1GA0017062*	54956054	1.75 × 10^−45^	24.9	
12	C18:2	92	42.1–59.6	*M1GA0017062*	54956054	3.78 × 10^−52^	26.5	
12	C18:3	2	53.0–53.7	*ASGA0055081*	53038625	9.38 × 10^−9^	6.4	
12	C20:0	2	54.9–55.5	*ASGA0055250*	55463919	1.95 × 10^−9^	5.4	
12	C20:1	1	56.9	*ASGA0055300*	56985632	1.17 × 10^−7^	4.4	
12	C20:4	71	46.6–59.6	*M1GA0017062*	54956054	2.66 × 10^−42^	29.4	
12	MUFA	65	44.7–59.6	*M1GA0017062*	54956054	9.88 × 10^−42^	23.3	
12	PUFA	97	42.1–59.7	*M1GA0017062*	54956054	1.01 × 10^−56^	28.9	
12	SFA	4	54.9–56.4	*H3GA0034916*	56324680	4.12 × 10^−8^	4.8	
12	UFA	10	54.9–58.9	*M1GA0017062*	54956054	3.25 × 10^−9^	5.6	
12	P/S ratio	88	42.1–59.6	*M1GA0017062*	54956054	9.93 × 10^−46^	24.8	
14	C16:1	3	111.9–119.1	*ALGA0081396*	119101432	4.79 × 10^−8^	5.4	*SCD*
14	C18:0	42	91.2–121.8	*H3GA0042070*	111950280	8.25 × 10^−19^	19.3	
14	SFA	3	110.5–111.9	*ALGA0081025*	110570159	1.04 × 10^−9^	9	
14	UFA	3	110.6–111.9	*ALGA0081025*	110570159	2.80 × 10^−10^	9.7	
16	C20:0	22	37.4–47.1	*ALGA0090392*	37382842	1.06 × 10^−14^	8.1	*ELOVL7*

^1^ Pig chromosome, ^2^ number of significant SNPs at the genome-wide level, ^3^ range of N_snp_, ^4^ genome map position on the *Sus scrofa* 11.1 genome assembly, ^5^ nominal *p*-values computed using the GEMMA package, ^6^ percentage of phenotypic variance explained by the top SNPs.

**Table 3:** Results of conditional association analyses for C18:1 and C18:2 using SNP markers in the 749.1 kb critical region in SSC12.

					C18:1				C18:2			
					Single-Marker Analysis		Conditional Analysis		Single-Marker Analysis		Conditional Analysis	
No	^1^ BP	SNP annotation	^2^ Allele 1	^3^ r	^4^ *b*	^5^*p*-value	^6^ *β*	^7^*p*-value	^4^ *b*	^5^*p*-value	^6^ *β*	^7^*p*-value
1	12:54812172	intron-*GAS7*	T	−0.443	−0.921	7.52 × 10^−^^07^	−0.035	0.852	0.108	5.53 × 10^−^^12^	0.030	0.059
2	12:54842795	intron-*GAS7*	T	0.390	0.443	0.038	−0.474	0.027	−0.056	0.0016	0.022	0.217
3	12:54901488	intron-*GAS7*	G	−0.382	−0.539	0.003	0.107	0.549	0.045	0.0024	−0.010	0.517
4	12:54929793	intron-*GAS7*	G	0.638	−1.380	3.24 × 10^−^^18^	−0.361	0.027	0.125	7.18 × 10^−^^21^	0.036	0.008
5	12:54956054	intron-*GAS7*	C	*-	2.301	2.45 × 10^−^^33^	**-	**-	−0.201	1.37 × 10^−^^35^	**-	**-
6	12:55073130	coding-*MYH13*	A	−0.581	−1.016	5.45 × 10^−^^8^	0.221	0.244	0.111	1.31 × 10^−^^12^	0.003	0.838
7	12:55093697	intron *MYH13*	A	−0.354	−0.478	0.020	0.247	0.229	0.031	0.0695	−0.031	0.076
8	12:55120479	3′UTR *MYH8*	G	−0.548	−1.347	9.75 × 10^−^^14^	−0.225	0.225	0.094	6.08 × 10^−^^10^	−0.0005	0.975
9	12:55180513	3′UTR *MYH4*	G	−0.556	−1.337	4.91 × 10^−^^14^	−0.236	0.194	0.095	2.08 × 10^−^^10^	0.002	0.906
**10**	**12:55229376**	**exon *MYH2***	**G**	**0.996**	**2.203**	**3.65 × 10^−^^31^**	**^†^ NA**	**^†^ NA**	**−0.192**	**3.42 × 10^−^^33^**	**^†^ NA**	**^†^ NA**
**11**	**12:55262653**	**exon *MYH2***	**T**	**0.996**	**2.251**	**3.91 × 10^−^^32^**	**^†^ NA**	**^†^ NA**	**−0.199**	**3.73 × 10^−^^35^**	**^†^ NA**	**^†^ NA**
12	12:55351879	intron *MYH3*	A	0.702	−1.449	1.57 × 10^−^^15^	0.031	0.871	0.146	9.72 × 10^−^^22^	0.017	0.280
13	12:55373617	5′UTR *MYH3*	G	0.866	1.865	3.54 × 10^−^^21^	−0.020	0.921	−0.189	7.08 × 10^−^^30^	−0.024	0.174
**14**	**12:55373707**	**5′UTR *MYH3***	**2**	**0.999**	**2.276**	**6.88 × 10^−^^33^**	**^†^ NA**	**^†^ NA**	**−0.198**	**4.85 × 10^−^^35^**	**^†^ NA**	**^†^ NA**
15	12:55447604	intron *ADPRM*	G	0.626	1.730	3.19 × 10^−^^12^	0.055	0.827	−0.141	1.31 × 10^−^^11^	0.002	0.922
16	12:55463919	intron *ADPRM*	G	0.946	2.252	7.42 × 10^−^^32^	0.119	0.560	−0.194	2.60 × 10^−^^33^	−0.008	0.637
17	12:55475542	intron *ADPRM*	T	−0.630	−1.329	1.03 × 10^−^^16^	−0.307	0.061	0.123	8.85 × 10^−^^20^	0.034	0.016
18	12:55530321	intron ENSSSCG 00000044652	A	−0.101	−1.559	2.87 × 10^−^^6^	−1.201	0.0004	0.065	0.0195	0.035	0.209
19	12:55561243	intron ENSSSCG 00000044652	T	0.106	0.378	0.022	0.205	0.216	−0.017	0.226	−0.002	0.883

^1^ Physical map position of SNP markers in the 749.1 kb critical region in SSC12; ^2^ reference allele; ^3^ LD correlation (r) between the key SNP marker (shaded with grey color; the SNP at 12:54956054) and corresponding SNP markers; ^4^ regression coefficients computed from single-marker analysis; ^5^ *p*-values computed from single-marker analysis; ^6^ regression coefficients calculated from conditional analysis; ^7^ *p*-values calculated from conditional analysis; bold characters represent SNP markers showing extremely high LD correlation with the key SNP marker; **^†^** absence of regression coefficient and significance due to collinearity originating from extremely high LD with the key SNP marker. * We did not calculate the LD correlation because r is the correlation between the key SNP and other SNPs; ** we did not compute the *β* and *p-*value of the key SNP marker in the conditional analysis.

**Table 4:** Results of conditional association analyses for C20:4 and MUFAs using SNP markers in the 749.1 kb critical region in SSC12.

					C20:4				MUFA			
					Single-Marker Analysis		Conditional Analysis		Single-Marker Analysis		Conditional Analysis	
No	^1^ BP	SNP annotation	^2^ Allele 1	^3^ r	^4^ *b*	^5^*p*-value	^6^ *β*	^7^*p*-value	^4^ *b*	^5^*p*-value	^6^ *β*	^7^*p*-value
1	12:54812172	intron-*GAS7*	T	−0.443	0.145	3.30 × 10^−^^11^	0.031	0.164	−1.021	4.32 × 10^−^^7^	0.204	0.694
2	12:54842795	intron-*GAS7*	T	0.390	−0.089	0.0003	0.025	0.324	0.543	0.0197	0.234	0.060
3	12:54901488	intron-*GAS7*	G	−0.382	0.076	0.0002	−0.004	0.861	−0.572	0.0033	0.195	0.537
4	12:54929793	intron-*GAS7*	G	0.638	0.182	2.24 × 10^−^^22^	0.050	0.010	−1.458	1.80 × 10^−^^17^	0.176	0.027
5	12:54956054	intron-*GAS7*	C	*-	−0.305	9.66 × 10^−^^42^	**-	**-	2.404	3.30 × 10^−^^31^	**-	**-
6	12:55073130	coding-*MYH13*	A	−0.581	0.154	1.43 × 10^−^^12^	−0.003	0.909	−1.108	5.52 × 10^−^^8^	0.191	0.354
7	12:55093697	intron *MYH13*	A	−0.354	0.059	0.014	−0.031	0.193	−0.479	0.032	0.294	0.189
8	12:55120479	3′UTR *MYH8*	G	−0.548	0.157	1.32 × 10^−^^13^	0.016	0.452	−1.389	1.59 × 10^−^^12^	−0.202	0.314
9	12:55180513	3′UTR *MYH4*	G	−0.556	0.160	1.19 × 10^−^^14^	0.022	0.310	−1.383	6.56 × 10^−^^13^	−0.222	0.260
**10**	**12:55229376**	**exon *MYH2***	**G**	**0.996**	**−0.289**	**2.77 × 10^−^^38^**	**^†^ NA**	**^†^ NA**	**2.302**	**3.74 × 10^−^^29^**	**^†^ NA**	**^†^ NA**
**11**	**12:55262653**	**exon *MYH2***	**T**	**0.996**	**−0.299**	**2.58 × 10^−^^40^**	**^†^ NA**	**^†^ NA**	**2.357**	**3.50 × 10^−^^30^**	**^†^ NA**	**^†^ NA**
12	12:55351879	intron *MYH3*	A	0.702	0.209	6.64 × 10^−^^23^	0.018	0.421	−1.613	3.45 × 10^−^^16^	−0.066	0.745
13	12:55373617	5′UTR *MYH3*	G	0.866	−0.272	8.39 × 10^−^^32^	−0.022	0.383	2.025	3.13 × 10^−^^21^	0.054	0.809
**14**	**12:55373707**	**5′UTR *MYH3***	**2**	**0.999**	**−0.301**	**5.67 × 10^−^^41^**	**^†^ NA**	**^†^ NA**	**2.381**	**7.35 × 10^−^^31^**	**^†^ NA**	**^†^ NA**
15	12:55447604	intron *ADPRM*	G	0.626	−0.228	3.51 × 10^−^^15^	−0.016	0.586	1.836	1.09 × 10^−^^11^	0.053	0.849
16	12:55463919	intron *ADPRM*	G	0.946	−0.291	5.64 × 10^−^^38^	−0.008	0.758	2.348	1.23 × 10^−^^29^	0.120	0.587
17	12:55475542	intron *ADPRM*	T	−0.630	0.178	4.09 × 10^−^^21^	0.045	0.020	−1.404	5.31 × 10^−^^16^	−0.328	0.064
18	12:55530321	intron ENSSSCG 00000044652	A	−0.101	0.127	9.33 × 10^−^^4^	0.084	0.030	−1.444	0.0001	−1.062	0.004
19	12:55561243	intron ENSSSCG 00000044652	T	0.106	−0.034	0.079	−0.012	0.523	0.376	0.036	0.193	0.285

^1^ Physical map position of SNP markers in the 749.1 kb critical region in SSC12; ^2^ reference allele; ^3^ LD correlation between the key SNP marker (shaded with grey color; the SNP at 12:54956054) and corresponding SNP markers; ^4^ regression coefficients computed from single-marker analysis; ^5^ *p*-values computed from single-marker analysis; ^6^ regression coefficients calculated from conditional analysis; ^7^ *p*-values calculated from conditional analysis; bold characters represent SNP markers showing extremely high LD correlation with the key SNP marker; **^†^** absence of regression coefficients and significance due to collinearity originated from extremely high LD with the key SNP marker. * We did not calculate the LD correlation because r is the correlation between the key SNP and other SNPs; ** We did not compute the *β* and *p-*value of the key SNP marker in the conditional analysis.

**Table 5:** Results of conditional association analyses for PUFA and P/S ratio using SNP markers in the 749.1 kb critical region in SSC12.

					PUFA				P/S Ratio			
					Single-Marker Analysis		Conditional Analysis		Single-Marker Analysis		Conditional Analysis	
No	^1^ BP	SNP annotation	^2^ Allele 1	^3^ r	^4^ *b*	^5^*p*-value	^6^ *β*	^7^*p*-value	^4^ *b*	^5^*p*-value	^6^ *β*	^7^*p*-value
1	12:54812172	intron-*GAS7*	T	−0.443	0.122	1.16 × 10^−^^12^	0.033	0.056	0.040	1.64 × 10^−^^9^	0.008	0.230
2	12:54842795	intron-*GAS7*	T	0.390	−0.066	0.0007	0.022	0.262	−0.020	0.0090	0.013	0.089
3	12:54901488	intron-*GAS7*	G	−0.382	0.056	0.0006	−0.006	0.723	0.014	0.0275	−0.009	0.160
4	12:54929793	intron-*GAS7*	G	0.638	0.141	7.24 × 10^−^^22^	0.039	0.010	0.052	1.92 × 10^−^^20^	0.016	0.007
5	12:54956054	intron-*GAS7*	C	*-	−0.233	1.03 × 10^−^^39^	**-	**-	−0.082	3.41 × 10^−^^33^	**-	**-
6	12:55073130	coding-*MYH13*	A	−0.581	0.127	1.08 × 10^−^^13^	0.005	0.769	0.042	5.65 × 10^−^^10^	−0.003	0.691
7	12:55093697	intron *MYH13*	A	−0.354	0.043	0.022	−0.027	0.153	0.010	0.157	−0.016	0.035
8	12:55120479	3′UTR *MYH8*	G	−0.548	0.112	1.58 × 10^−^^11^	0.004	0.795	0.042	1.11 × 10^−^^10^	0.002	0.743
9	12:55180513	3′UTR *MYH4*	G	−0.556	0.113	3.81 × 10^−^^12^	0.007	0.665	0.041	8.68 × 10^−^^11^	0.002	0.712
**10**	**12:55229376**	**exon *MYH2***	**G**	**0.996**	**−0.222**	**6.45 × 10^−^^37^**	**^†^ NA**	**^†^ NA**	**−0.078**	**1.89 × 10^−^^30^**	**^†^ NA**	**^†^ NA**
**11**	**12:55262653**	**exon *MYH2***	**T**	**0.996**	**−0.231**	**4.27 × 10^−^^39^**	**^†^ NA**	**^†^ NA**	**−0.080**	**3.13 × 10^−^^32^**	**^†^ NA**	**^†^ NA**
12	12:55351879	intron *MYH3*	A	0.702	0.163	1.18 × 10^−^^22^	0.015	0.376	0.060	3.49 × 10^−^^20^	0.007	0.287
13	12:55373617	5′UTR *MYH3*	G	0.866	−0.215	3.23 × 10^−^^32^	−0.024	0.225	−0.075	1.58 × 10^−^^26^	−0.008	0.280
**14**	**12:55373707**	**5′UTR *MYH3***	**2**	**0.999**	**−0.230**	**4.66 × 10^−^^39^**	**^†^ NA**	**^†^ NA**	**−0.081**	**1.07 × 10^−^^32^**	**^†^ NA**	**^†^ NA**
15	12:55447604	intron *ADPRM*	G	0.626	−0.166	2.98 × 10^−^^13^	−0.004	0.877	−0.060	1.21 × 10^−^^11^	−0.0003	0.972
16	12:55463919	intron *ADPRM*	G	0.946	−0.224	7.37 × 10^−^^37^	−0.008	0.667	−0.080	9.11 × 10^−^^32^	−0.004	0.548
17	12:55475542	intron *ADPRM*	T	−0.630	0.139	9.59 × 10^−^^21^	0.036	0.018	0.051	2.38 × 10^−^^19^	0.015	0.013
18	12:55530321	intron ENSSSCG 00000044652	A	−0.101	0.082	6.51 × 10^−^^3^	0.049	0.108	0.035	0.0036	0.022	0.066
19	12:55561243	intron ENSSSCG 00000044652	T	0.106	−0.021	0.167	−0.004	0.779	−0.008	0.160	−0.002	0.716

^1^ Physical map position of SNP markers in the 749.1 kb critical region in SSC12; ^2^ reference allele; ^3^ LD correlation between the key SNP marker (shaded with grey color; the SNP at 12:54956054) and corresponding SNP marker; ^4^ regression coefficients computed from single-marker analysis; ^5^ *p*-values computed from single-marker analysis; ^6^ Regression coefficients calculated from conditional analysis; ^7^ *p*-values calculated from conditional analysis; bold characters represent SNP markers showing extremely high LD correlation with the key SNP marker. **^†^** Absence of regression coefficients and significance due to collinearity originated from extremely high LD with the key SNP marker. * We did not calculate the LD correlation because r is the correlation between the key SNP and other SNPs; ** We did not compute the *𝛃* and *p*-value of the key SNP marker in the conditional analysis.

## Data Availability

Restrictions apply to the availability of these data. Data were obtained from the National Institute of Animal Science (NIAS), Republic of Korea, and are available with the permission of the NIAS.

## Supplementary Materials

The following are available online at https://www.mdpi.com/article/10.3390/genes12091323/s1, Supplementary Table S1: Haplotype and diplotype frequencies of the GAS7, MYH2, and MYH3 genes in the LK F2 pigs; Supplementary Figure S1: Quantile–Quantile plots (qq plots) of the GWAS for FA composition traits in the LK cross; Supplementary Figure S2: The 749.1 kb critical region in SSC12 identified by the LALD mapping. SNP position is the physical base pair (bp) position in SSC12 (Sus scrofa 11.1); Supplementary Figure S3: Regional *p*-value plots obtained from the single-marker association analysis using GEMMA for the six FA profile traits.
