# Supplementary material for: GWAS and Post-GWAS High-Resolution Mapping Analyses Identify Strong Novel Candidate Genes Influencing the Fatty Acid Composition of the Longissimus dorsi Muscle in Pigs
Source: Genes (Basel). 2021 Aug 26;12(9):1323. doi: 10.3390/genes12091323 (PMC8468772; doi:10.3390/genes12091323)
Supplement: Supplementary file 1 [file genes-12-01323-s001.zip › genes-1348889-supplementary.pdf]

Supplementary Materials

Supplementary Table S1. Haplotype and diplotype frequencies of the GAS7, MYH2 and MYH3 genes in the LK F_2 pigs.

[1]Haplotype	Number	[2]Frequency	Diplotype	Number	Frequency
T-A-C-1 (ht1)	1562	0.71	ht1/ht1	565	0.51
C-G-T-2 (ht2)	641	0.29	ht1/ht2	425	0.39
			ht2/ht2	108	0.10

[1]The 1st locus is *GAS7* g.18482 T>C (12:54,956,054); the 2nd locus is *MYH2* c.-449935 A>G (12:55,229,376); the 3rd locus *MYH2* c.–22675 C>T (12:55,262,653) and the 4th locus is *MYH3*–1805_–1810delCAGTCC (15:55,373,707), [2]The haplotypes with frequency less than 0.001 are dropped in this table.

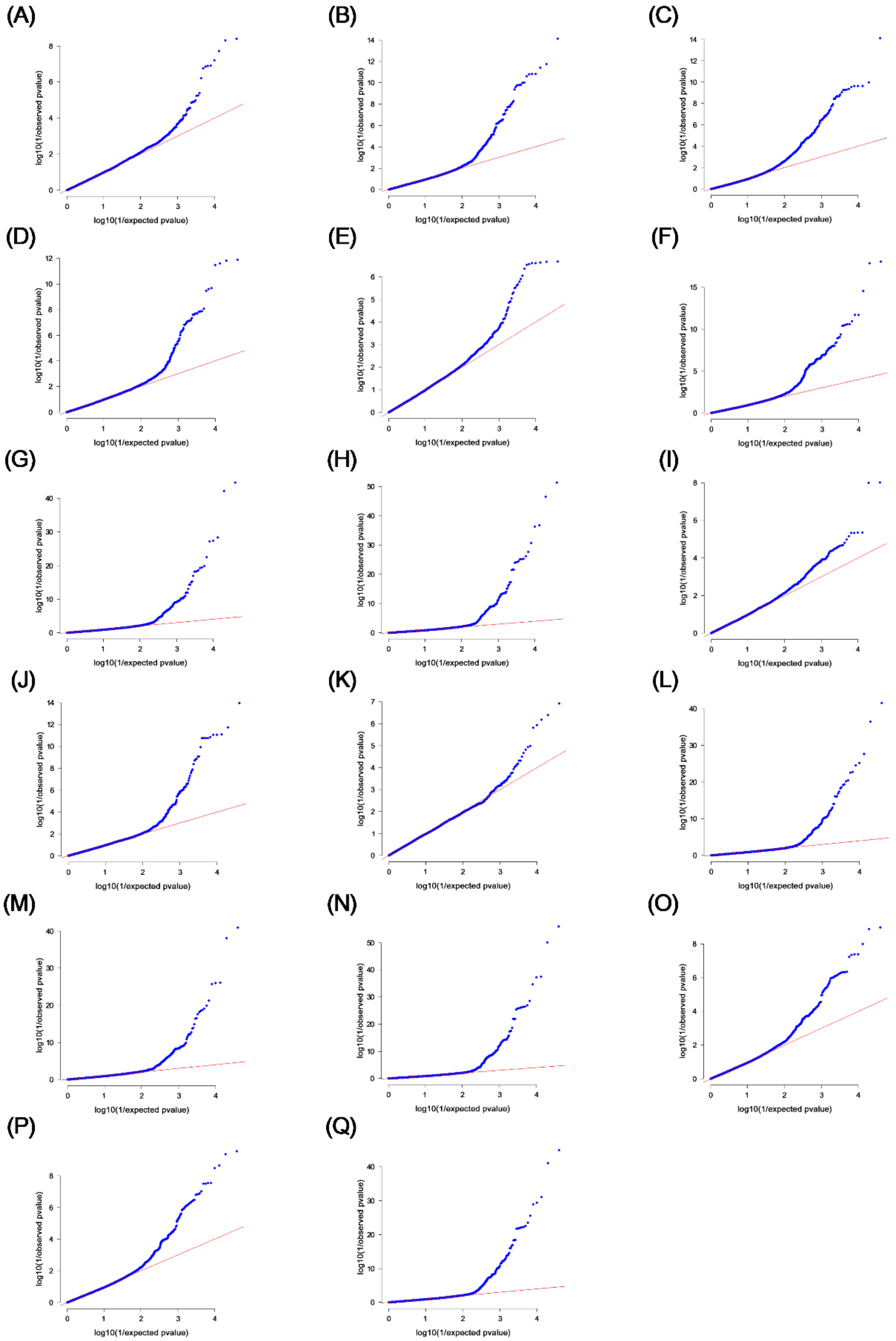

Supplementary Figure S1. Quantile–Quantile plots (qq plots) of the GWAS for FA composition traits in the LK cross. (A) QQ plot for C12:0; (B) QQ plot for C16:0; (C) QQ plot for C16:1; (D) QQ plot for C17:0; (E) QQ plot for C17:1; (F) QQ plot for C18:0; (G) QQ plot for C18:1; (H) QQ plot for C18:2; (I) QQ plot for C18:3; (J) QQ plot for C20:0; (K) QQ plot for C20:1; (L) QQ plot for C20:4; (M) QQ plot for MUFAs; (N) QQ plot for PUFA; (O) QQ plot for SFA; (P) QQ plot for UFA; (Q) QQ plot for P/S ratio. The genomic inflation factor (λ) values ranged from 0.78 to 0.94 for all 17 results of GWAS.

SNP annotation	SNP position (BP)	LOD_C18:1	LOD_C18:2	LOD_C20:4	LOD_MUFA	LOD_PUFA	LOD_P/S ratio	LOD_C12:0	LOD_C16:0	LOD_C17:0	LOD_C17:1	LOD_C18_3	LOD_C20_0	LOD_C20_1	LOD_UFA	LOD_SFA
	53870191	38.4	48.5	55.1	33.7	53.8	40.6	2.4	15.2	10.8	5.7	7.0	7.5	6.7	10.0	8.4
	53913684	38.4	48.5	55.1	33.7	53.8	40.6	2.4	15.2	10.8	5.7	7.0	7.5	6.7	10.0	8.4
	53945418	38.4	48.5	55.1	33.7	53.8	40.6	2.4	15.2	10.8	5.7	7.0	7.5	6.7	10.0	8.4
	53984115	37.4	47.7	53.9	32.7	52.9	40.0	2.4	15.1	10.6	5.7	6.9	7.6	6.6	10.0	8.5
	54047606	36.9	47.0	53.7	32.2	52.2	39.5	2.4	14.6	10.2	5.5	6.4	7.7	6.0	9.9	8.4
	54199661	46.4	56.7	67.7	41.0	63.5	50.0	3.0	17.4	12.0	6.7	7.1	10.5	7.3	12.6	10.6
	54201422	46.4	56.7	67.7	41.0	63.5	50.0	3.0	17.4	12.0	6.7	7.1	10.5	7.3	12.6	10.6
	54203411	46.4	57.0	68.3	41.1	63.9	50.4	3.0	17.3	11.8	6.7	7.1	10.6	7.2	12.6	10.6
	54215585	46.4	57.0	68.3	41.1	63.9	50.4	3.0	17.3	11.8	6.7	7.1	10.6	7.2	12.6	10.6
	54252731	46.4	57.0	68.3	41.1	63.9	50.4	3.0	17.3	11.8	6.7	7.1	10.6	7.2	12.6	10.6
	54264915	46.4	57.0	68.3	41.1	63.9	50.4	3.0	17.3	11.8	6.7	7.1	10.6	7.2	12.6	10.6
	54307554	46.4	57.0	68.3	41.1	63.9	50.4	3.0	17.3	11.8	6.7	7.1	10.6	7.2	12.6	10.6
	54360843	46.4	57.0	68.3	41.1	63.9	50.4	3.0	17.3	11.8	6.7	7.1	10.6	7.2	12.6	10.6
intron-GAS7	**54812172**	50.6	62.0	73.2	46.5	69.8	54.4	3.1	18.9	12.4	7.3	8.0	10.5	8.8	13.3	11.0
intron-GAS7	**54842795**	52.5	63.8	74.9	46.9	71.4	55.3	3.0	18.7	12.9	7.6	8.0	10.6	8.8	12.6	10.4
intron-GAS7	**54901488**	52.5	63.8	74.9	46.9	71.4	55.3	3.0	18.7	12.9	7.6	8.0	10.6	8.8	12.6	10.4
intron-GAS7	**54929793**	52.5	63.8	74.9	46.9	71.4	55.3	3.0	18.7	12.9	7.6	8.0	10.6	8.8	12.6	10.4
intron-GAS7 (g.18482 C/T)	**54956054**	52.7	63.9	75.1	47.1	71.7	55.4	3.0	18.8	12.9	7.6	8.0	11.0	8.8	12.7	10.5
coding-MYH13	**55073130**	52.7	63.9	75.1	47.1	71.7	55.4	3.0	18.8	12.9	7.6	8.0	11.0	8.8	12.7	10.5
intron-MYH13	**55093697**	52.7	63.9	75.1	47.1	71.7	55.4	3.0	18.8	12.9	7.6	8.0	11.0	8.8	12.7	10.5
intergenic-MYH13_5'-MYH8_3'	**55120479**	52.7	63.9	75.1	47.1	71.7	55.4	3.0	18.8	12.9	7.6	8.0	11.0	8.8	12.7	10.5
intergenic-MYH8_5'-MYH4_3'	**55180513**	52.7	63.9	75.1	47.1	71.7	55.4	3.0	18.8	12.9	7.6	8.0	11.0	8.8	12.7	10.5
intron-MYH1	**55229376**	52.7	63.9	75.1	47.1	71.7	55.4	3.0	18.8	12.9	7.6	8.0	11.0	8.8	12.7	10.5
exon-MYH1	**55262653**	52.7	63.9	75.1	47.1	71.7	55.4	3.0	18.8	12.9	7.6	8.0	11.0	8.8	12.7	10.5
MYH3-5'UTR	**55373617**	52.9	64.2	75.0	47.3	71.9	55.4	2.9	18.7	13.0	7.8	8.2	11.4	8.8	12.6	10.4
intron-ADPRM	**55463919**	52.1	62.9	73.5	46.6	70.4	54.4	2.9	18.4	12.8	7.7	8.1	11.1	8.6	12.4	10.3
intron-ADPRM	**55475542**	52.1	62.9	73.5	46.6	70.4	54.4	2.9	18.4	12.8	7.7	8.1	11.1	8.6	12.4	10.3
PIRT-5'UTR	**55530321**	52.1	62.9	73.5	46.6	70.4	54.4	2.9	18.4	12.8	7.7	8.1	11.1	8.6	12.4	10.3
PIRT-5'UTR	**55561243**	52.1	62.9	73.5	46.6	70.4	54.4	2.9	18.4	12.8	7.7	8.1	11.1	8.6	12.4	10.3
	55636692	44.9	56.2	64.1	40.5	62.7	48.5	2.7	16.5	12.0	6.9	6.7	10.1	7.1	11.5	9.5
	55706702	44.9	56.2	64.1	40.5	62.7	48.5	2.7	16.5	12.0	6.9	6.7	10.1	7.1	11.5	9.5
	55721930	44.9	56.2	64.1	40.5	62.7	48.5	2.7	16.5	12.0	6.9	6.7	10.1	7.1	11.5	9.5
	55856415	44.2	55.7	63.6	39.9	62.2	48.0	2.7	16.3	11.7	6.8	6.5	10.1	6.9	11.3	9.4
	56016099	44.2	55.7	63.6	39.9	62.2	48.0	2.7	16.3	11.7	6.8	6.5	10.1	6.9	11.3	9.4
	56060292	43.9	55.4	63.2	39.5	61.7	47.7	2.7	16.2	11.6	6.8	6.5	10.0	6.9	11.3	9.4
	56076247	43.9	55.4	63.2	39.5	61.7	47.7	2.7	16.2	11.6	6.8	6.5	10.0	6.9	11.3	9.4
	56269132	43.8	55.1	63.0	39.4	61.4	**47.7**	2.6	16.0	11.5	6.7	6.5	10.0	7.0	11.1	9.1

749.1-kb shared region of linkage and linkage disequilibrium (SSC12: 54,812,172 -55,561,243)

Supplementary Figure S2. The 749.1-kb critical region in SSC12 identified by the LALD mapping. SNP position is the physical base pair (BP) position in SSC12 (*Sus scrofa* 11.1). LOD represents the LOD score for each FA profile trait. The red-colored values represent maximum LOD scores for the FA composition trait in the LK cross. The 1-LOD drop confidence interval was applied to each FA profile traits (orange color). The black box line is highlights the 749.1-kb critical region shared across the traits of interest. .

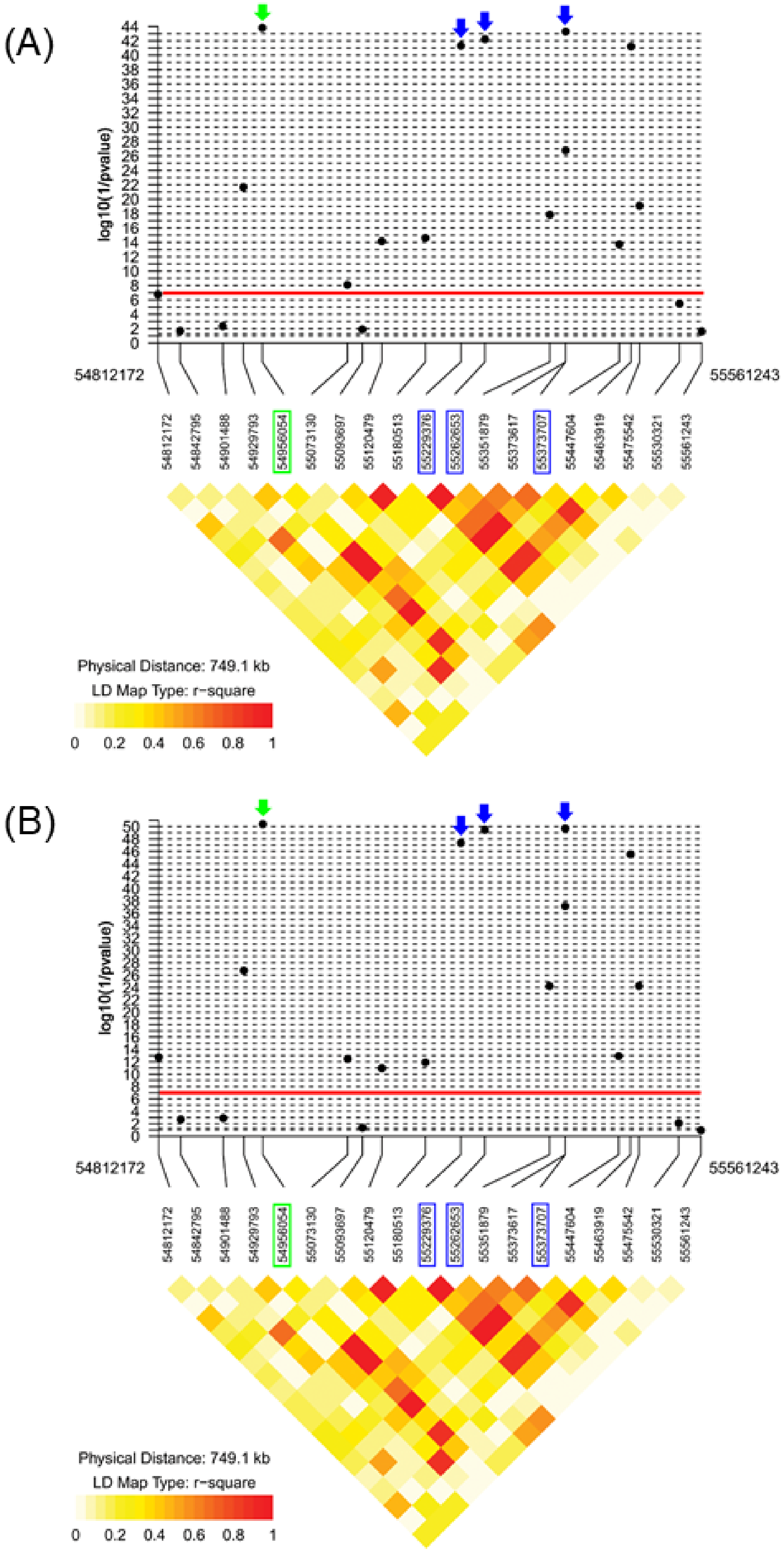

Supplementary Figure S3. Continued.

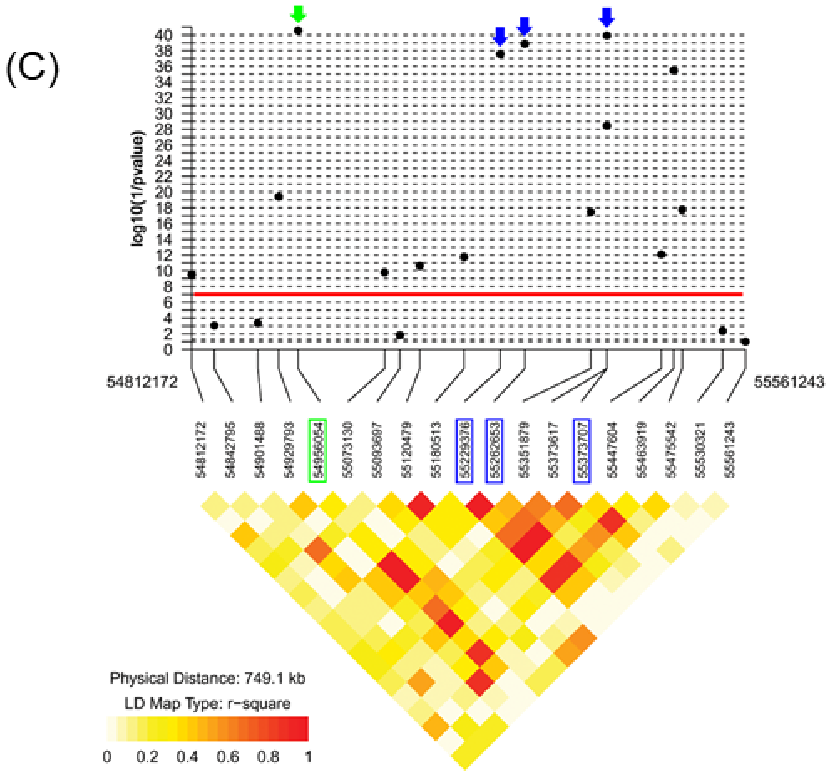

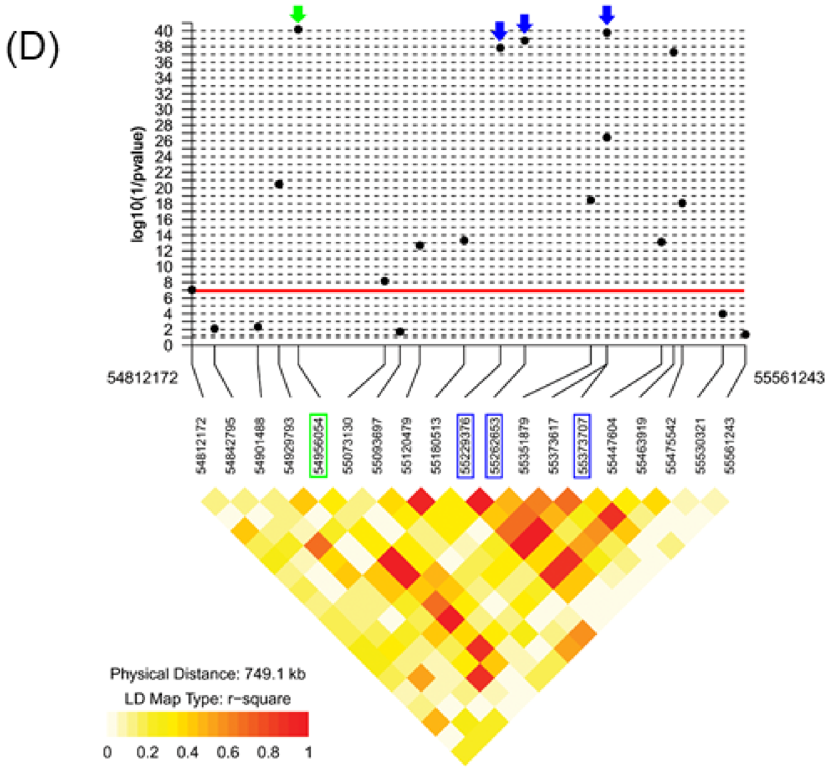

Supplementary Figure S3. Continued.

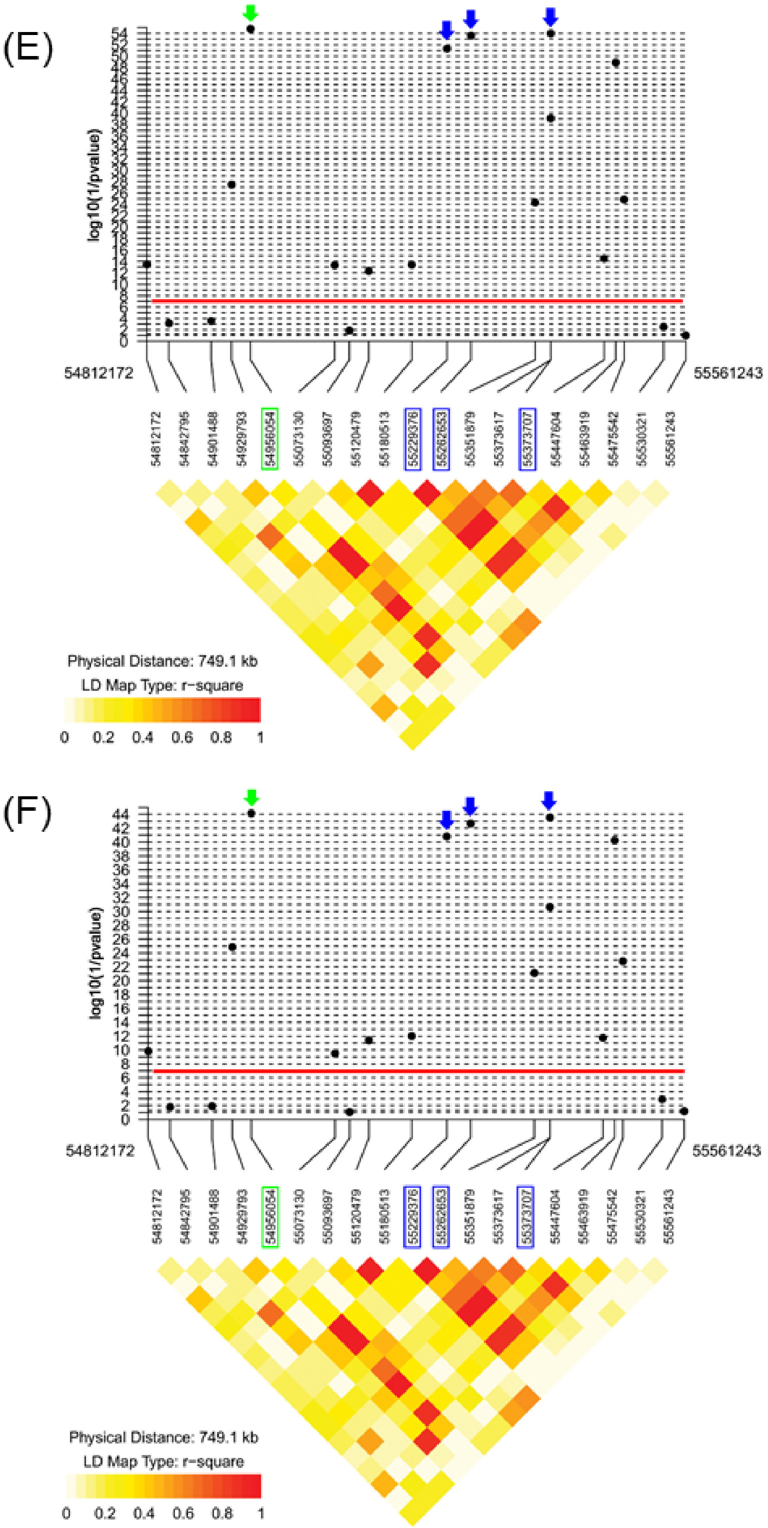

Supplementary Figure S3. Regional *p*-value plots obtained from the single-marker association analysis using GEMMA for the six FA profile traits. The y-axis shows the $\log_{10}$(*p*-value), and the x-axis shows the physical positions of the SNP markers in SSC12. The genome-wide significance threshold value is 6.60, which equals Bonferroni's correction of 1% (represented by the red horizontal lines). There are lines to connect the pairwise LD structure with a black horizontal line representing the 749.1-kb critical region. The genome map position of each SNP marker is demonstrated above the LD plot. The light green colored box indicates the position of *GAS7:g.18482T>C*. The magnitude of LD by r-square statistic is shown. The light green colored arrow in each panel indicates the significance of the key SNP marker identified by GCTA-slct procedure. The blue colored arrows and boxes in each panel indicates significance levels of candidate genes identified by conditional association analysis and their locations. (A) Regional *p*-value plot for C18:1; (B) Regional *p*-value plot for C18:2; (C) Regional *p*-value plot for C20:4; (D) Regional *p*-value plot for MUFAs (E) Regional *p*-value plot for PUFAs; (F) Regional *p*-value plot for P/S ratio.
